# Experimental investigation of laminar and turbulent displacement of residual oil film

**DOI:** 10.1038/s41598-023-48563-x

**Published:** 2023-11-30

**Authors:** Yao Zhang, Benjamin Barrouillet, Hans Joakim Skadsem

**Affiliations:** Department of Energy and Petroleum Engineering, University of Stavanger, Stavanger, 4068 Norway

**Keywords:** Environmental sciences, Engineering, Applied physics, Fluid dynamics, Techniques and instrumentation

## Abstract

Residual oil films on pipe walls are a common occurrence in industrial processes, and their presence can significantly impact system efficiency and performance. However, the mechanisms that govern oil film removal by an immiscible displacing fluid from the internal walls of pipes under different flow regimes, including laminar and turbulent flows, are not yet fully understood. In this study, we investigated the impact of displacing fluid flow regime, injected volume, displacement time, and wall shear stress on the efficiency of residual oil film removal in a pipe. We first verified the applicability of our developed oil film measurement method for the use in vertical pipes, and found that gravity did not significantly affect the long-term oil film removal process. We verified that our results from the laminar cases agree with the theoretical thin-film limit scaling under reasonable assumptions of constant shear stress and negligible surface tension. We then examined the displacement efficiency of residual oil film under laminar and turbulent flow regimes. Our experimental results revealed that the onset of turbulence of displacing fluid played an important role in the efficient removal of residual oil film, with an optimal range of Reynolds numbers (7000–8000) when the injected volume of displacing fluid is limited. Furthermore, we explored the combined effect of wall shear stress and displacement time on the displacement process under different turbulent flow regimes. We found that the intermediate turbulent regime was the most efficient for achieving cleaning in a limited time, while the highly turbulent regime proved to be the most effective for achieving complete cleaning over a longer time period. These findings have important implications for oil recovery and pipeline maintenance and provide valuable insights into optimizing the removal of residual oil film in pipes.

## Introduction

Fluid mechanical cleaning is a critical operation within various industries, such as the medical, pharmaceutical, and cosmetic sectors. The efficiency of cleaning operations directly affects factors such as energy consumption, liquid and chemical usage, and product quality^1,2^. Failure to adequately clean and decontaminate surfaces can lead to cross-contamination of liquids, posing significant risks to health, safety, and the environment^3^. Effective fluid displacement and surface cleaning are also essential for the construction of wells for geological carbon storage and geothermal energy recovery^4^. Well construction involves multiple stages, with each section completed by running a casing string to the bottom of the wellbore and placing a cement slurry in the annular space between the casing and the newly drilled formation. Once hardened, the primary function of well cement is to enable zonal isolation along the well and prevent uncontrolled fluid flow along the annulus, which may pose safety and environmental risks^4^.

To achieve a low-permeable cement barrier capable of fulfilling its primary function, the drilling fluid initially occupying the annular space must be displaced, and the annular walls must be cleaned to allow proper bonding between the cement, casing, and formation. To facilitate the removal and cleaning process, a sequence of cementing fluids, including water-based washes or spacer fluids and the cement slurry, is pumped down the well. The cementing fluids first displace the core of the annulus, leaving behind thin films of drilling fluid along the walls, which are gradually removed through a combination of fluid mechanical, chemical, and surfactant cleaning mechanisms^2–4^. For the well construction and cementing application in particular, displacing fluids can flow in either laminar or turbulent regimes. Indeed, a combination of low-viscosity washing fluids flowing in turbulent regime, preceeded and/or followed by more viscous and denser spacer fluids in laminar flow is often recommended^4^. Design of effective laminar displacements typically rely on maintaining a *stable* density and viscosity hierarchy between displaced and displacing fluid, i.e. minimizing the inter-mixing between the fluids by ensuring the displacing fluid is denser and more viscous than the fluid to be displaced. This design seeks to prevent interface instabilities, such as Rayleigh–Taylor or Saffman–Taylor instabilities, and promote a stable, piston-like displacement of the core fluid. The purpose of low-viscosity washing fluids is to ensure fluid removal from walls by a combination of mechanisms, including erosion of wall film by shear instabilities and fluctuations, and thinning of the wall film liquid by dispersants added to the washing fluid^3,4^. For turbulent displacements in particular, the industry often refers to *minimum contact times* required to complete fluid displacement; since shear-driven erosion and the action of dispersants clean gradually, and not instantaneously, contact time recommendations ranging from a few minutes to 10 minutes or more^5^. Following such recommendations, the required volume of displacing washing fluid is determined by combining the imposed flow rate with the recommended contact time (i.e. duration of “contact” between the fluids).

Although fluid displacements in the context of well cementing has been studied extensively in the past, most such work have focused on core displacement mechanics involving miscible liquids. Indeed, the above-mentioned contact time recommendations for complete wall cleaning remain largely empirical, and driven by past experience and not a mechanical or physical modelling of the cleaning process^4^. Comparatively fewer studies have addressed fluid removal from walls, or displacement involving immiscible liquids in pipe or annular geometries. There is also a lack of available datasets that can be used for testing theoretical or numerical models for predicting wall film removal. The objective of the current study is to utilize a novel experimental methodology that allows accurate determination of very small residual fluid volumes^7^, and compare cleaning efficiency of laminar and different turbulent flowing conditions. We address displacement and wall film removal involving immiscible liquids, and study the residual oil film volume by staining the oil by a known concentration of the hydrophobic dye Nile red. The methodology is inspired by recent experiments that considered Couette-driven flow, and that showed that wettability, metallurgy and wall roughness all can impact the film removal rate^8,9^. We intend the current study to complement past work on immiscible core displacements^10,11^, and improve our understanding of how shear stress and displacement time impact the cleaning rate.

## Experiments and methods

### Experimental setup

**Figure 1:**
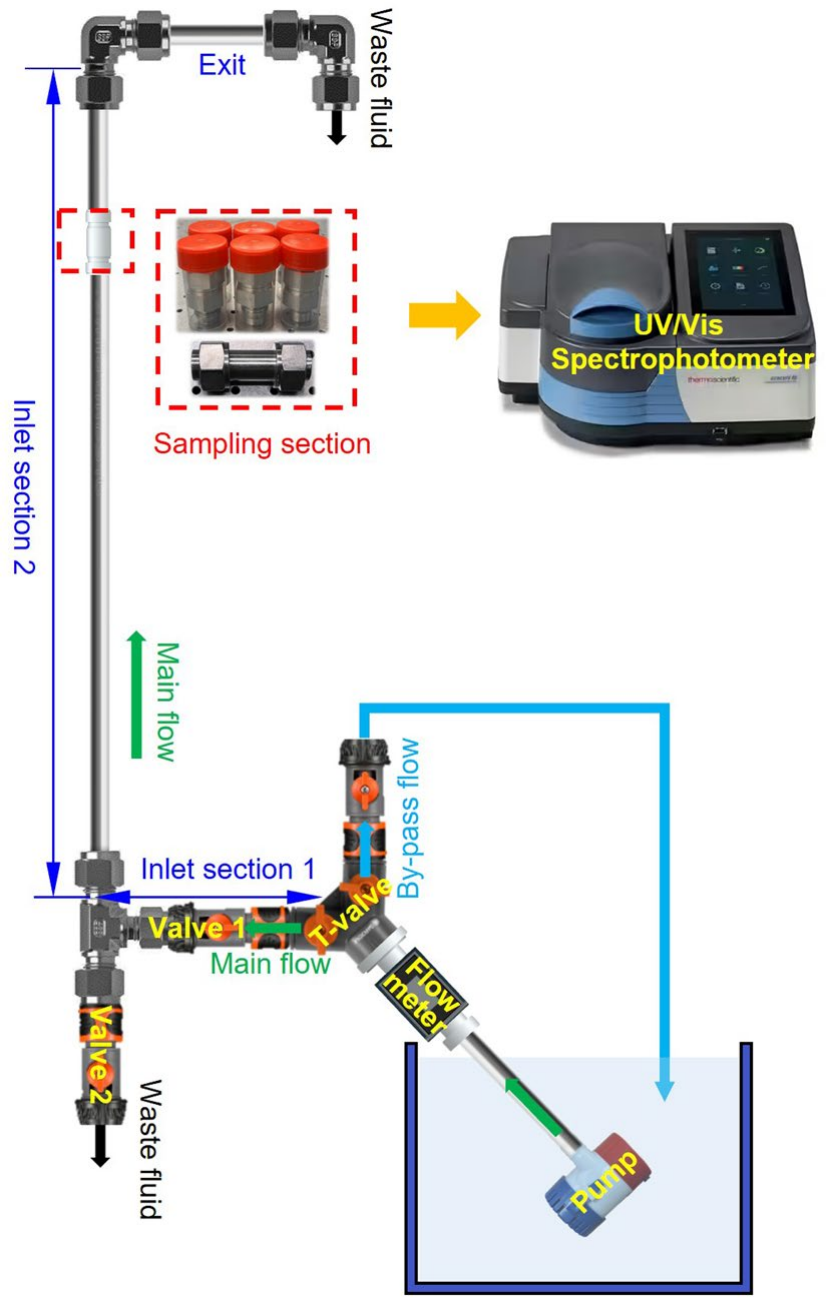
Schematic of the experimental setup.

The experimental setup used in this study was shown in Fig. 1 and was a modified version of the setup previously documented in^7^. The modification involved rotating the setup from a horizontal to a vertical orientation in order to align gravity parallel to the axis of the pipe. As we study pipe displacement involving a density-stable fluid hierarchy (denser displacing fluid injected from below), having the pipe vertical should prevent buoyancy-induced stratification of the interface.

As shown in Fig. 1, the setup consisted of connected stainless steel pipe segments: An inlet section 1 measuring 30 cm, an inlet section 2 measuring 65 cm, a sampling section measuring 7 cm, and an exit section. All pipe segments were constructed from stainless steel 316 and had an inner diameter of 15.05 mm. The arithmetic average roughness of the inner pipe wall was measured to be approximately 0.4 $\upmu$m using a portable surface roughness tester (SURFTEST SJ-210 SERIES, Mitutoyo Ltd.). An important function of inlet section 2 was to allow flow development from the inlet to the sampling section. The length of section 2 was equivalent to approximately 43 hydraulic diameters, which was assumed sufficient to ensure fully developed turbulent flow and to minimize entrance effects. We note that this length was likely not sufficiently long to ensure fully developed flow for the *laminar* cases considered in this study.

Fluid injection was performed using a centrifugal pump controlled by an external power supply, while the corresponding flow rate was measured using an ultrasonic flow meter (SICK Sensor Intelligence Model FFUS15-1G1IO) that was installed between the pump and inlet section 1, as shown in Fig. 1. The pump delivered the displacing fluid to the bottom of the pipe and pumped fluids upward, in the opposite direction of gravity. To prevent any pressure and flow rate changes during the displacement process, the outlet was designed to be open, allowing fluids to flow freely out of the system to an effluent collection tank. A by-pass line that allowed recirculation of displacing fluid into the main fluid container before displacement commenced was installed directly downstream of the flow meter.

All experiments reported in this study used a low-viscosity mineral oil (Sipdrill 4/0) as displaced fluid, and tap water as displacing fluid. The properties of these fluids, including density, viscosity, and interfacial tension, are shown in Table 1.

**Table 1:** Fluid properties.

Fluid	Density (kg/m$^3$)	Viscosity (mPa s)	Interfacial tension (mN/m)
Sipdril 4/0 (oil)	820	2.95	44.7
Water	998	1.00	

### Experimental procedures

Experiment preparation began by filling each section of the pipe with the appropriate fluids. Inlet section 1 was initially filled with displacing fluid (water), including the section between the bottom valve shown in Fig. 1 and inlet section 1. Inlet section 2 and the sampling section were filled with the displaced fluid (oil). This ensured that the displacement process started at the correct position. After filling the different sections of the pipe with the appropriate fluids, we waited for a period of 30 s to allow for the neutral separation of the immiscible fluids before starting the displacement process. It is important to note that the valve 1 and the valve 2 were initially closed to isolate fluids in the pipe, and valve 2 only opened at the end of each experiment to empty the fluid in the pipe. On the other hand, the sampling section and its neighboring sections needed to be properly cleaned before starting the experimental procedure.

Next, water was pumped through the by-pass line at the target flow rate for the experiment. To initiate the displacement process, the bypass valve was closed, and valve 1 was opened simultaneously. The displacing fluid would next push the displaced oil towards the outlet at the top of the pipe, where it was collected in the waste collector. Each displacement experiment was performed by injecting displacing fluid at a fixed flow rate, and for a predetermined duration.

At the end of each experiment, when the desired injected volume or time had been reached, the main flow loop was closed while the bypass loop was opened. The remaining fluid in the pipe was then emptied through the valve 2, and the sampling collection section was retrieved and replaced with a new one for the next experiment.

### Test matrix

Displacement experiments were performed at different imposed flow rates, *Q*, and using different volume of injected, displacing fluid. An overview of the experiments is provided in Table 2. For all cases listed in the table, experiments were performed by injecting 1 liter (L) and 2 L of total displaced fluid volume. In the table, the *displacement time* (i.e. experiment duration) corresponding to injection of 1 L displacing fluid is listed.

Also listed in the table is the Reynolds number, *Re*, which we define as $Re = {\rho }_w {U} {D} / {\mu }_w$ for the displacing fluid, i.e. water. Here, $\rho _w$ and $\mu _w$ denote the mass density and viscosity of water, and *D* denotes the inner pipe diameter, while $U = Q/(\pi D^2/4)$ is the imposed bulk velocity. In the table we also list the corresponding Reynolds number for the displaced fluid, i.e. oil, for completeness. In the following, we will however categorize displacements according to the Reynolds number of the *displacing* fluid only, i.e. water.

**Table 2:** Overview of flow rates and flowing conditions for the displacement experiments.

Case #	Flow rate(L/min)	Bulk velocity (m/s)	Displacement time, 1 L (s)	*Re*		Flow regime
				Oil	Water	
1	1.05	0.10	57	412	1480	Laminar
2	1.45	0.14	41	568	2044	Laminar
3	3.5	0.33	17	1372	4935	Turbulent
4	4.3	0.40	14	1685	6063	Turbulent
5	5	0.47	12	1960	7050	Turbulent
6	5.8	0.54	10	2273	8178	Turbulent
7	7	0.66	9	2744	9870	Turbulent
8	9	0.84	7	3527	12690	Turbulent

The experiments covered a range of Reynolds numbers in laminar and turbulent regimes, where we categorize the experiments with $Re < 2300$ as *laminar* displacements, and experiments with $Re > 4000$ as *turbulent*, based on the displacing fluid Reynolds number. To better distinguish between laminar and turbulent regimes, intermediate Reynolds numbers between 2300 and 4000 were not included in the current test program. As pointed out above, displacement experiments corresponding to the configurations specified in Table 2 were performed by injecting a fixed volume of 1 L or 2 L of water into the circular pipe. The 1 L of injected volume corresponded to approximately 6.5 times the oil volume initially filling the pipe, which was 155 ml.This volume was chosen based on previous experience, and to enable highly accurate comparison with previous work^7^.

Next, to investigate the effect of displacement time, or equivalently different injection volumes, on the removal of residual oil film in turbulent flow regime, three turbulent cases were selected for further study: low turbulent (Case 3, $Re = 4935$), intermediate turbulent (Case 5, $Re = 7050$), and high turbulent (Case 8, $Re = 12{,}690$), as per Table 2. For each of these cases, we varied the injected volume of water according to Table 3.

**Table 3:** Injected water volumes for turbulent cases.

Case #	Flow rate (L/min)	*Re*	Injected water volumes
3	3.5	4935	1, 1.5, 2, 2.5, 3, 3.5, 3.85
5	5	7050	1, 1.5, 2, 2.5, 3, 3.5, 3.8
8	9	12690	1, 1.5, 2, 2.5, 3, 3.5, 3.25

We end the presentation of the experimental test matrix by referring to the thin-film evolution equation derived by Oron et al.^12^, and that applies for the wall film within the lubrication approximation. Denoting by *h* the wall film thickness, the corresponding one-dimensional evolution equation for a liquid film adhering to a vertical wall and subject to a constant tangential shear stress $\tau$ at the film interface^12^, is:

1 $$\begin{aligned} \mu _o \partial _t h + \tau h \partial _x h + (\rho _w - \rho _o)g h^2 \partial _xh + \frac{1}{3} \sigma \partial _x (h^3 \partial _x^3 h) = 0. \end{aligned}$$

Here, *x* is in the upward vertical direction, *g* denotes the gravitational acceleration and $\sigma$ is the interfacial tension at the film interface. In the thin-film limit, the flux term due to buoyancy (third term) is seen to be of higher order than the constant forcing due to the shear stress at the interface (second term). Consequently, we anticipate (for thin films) that interface evolution is mainly driven by the shear stress exerted by the displacing fluid^12,13^. Now, balancing the first and second terms, we note that a characteristic time scale of the displacement may be taken proportional to $\mu _o / \tau$. Consequently, we will in the result section that follows discuss residual oil volume as functions of both displacement time, total injected volume and, motivated by Eq. 1, a dimensionless time $T = \tau _w \cdot t / \mu _o$, where *t* is the duration (or displacement time) of the experiment.

The fact that Eq. 1 was derived by Oron *et al.*^12^ for a plane channel geometry does not alter the identification of relevant time scale for the displacement experiments, nor the order at which buoyancy impacts the film evolution^13^. In what follows, we will approximate the shear stress at the interface, $\tau$, by the *wall* shear stress evaluated for the displacing fluid. That is, $\tau \approx \tau _w = 8 \mu _w U / D$ for laminar displacements, and with $\tau \approx \tau _w = \rho U^2 f_D / 8$ for turbulent displacements, where we invoke the Swamee-Jain friction factor^14^:

2 $$\begin{aligned} f_D = \frac{0.25}{\left[ \log _{10}\left( \frac{\frac{\varepsilon }{D}}{3.7} + \frac{5.74}{\text {Re}^{0.9}}\right) \right] ^2}. \end{aligned}$$

Here $\varepsilon$ is the pipe wall roughness, which we set to zero when evaluating the shear stress along the (assumed) smooth fluid-fluid interface. We also note that the above approximations likely overestimate the actual interfacial shear stress due to the stream-wise flow inside the film layer.

### Measurement and calculation of residual oil volume

A detailed explanation of the experimental methodology and analysis used to measure residual oil volume after displacement was provided in our previous study on the topic^7^.

To summarize, the residual oil volume at the end of each displacement experiment can be measured by first staining the oil with a known amount of hydrophobic dye, Nile red. At the end of the displacement experiment, the residual oil volume inside the sampling section is recovered by submerging this section in a fixed volume of tetrahydrofuran (THF), a water-miscible organic solvent, which produces a solution with a certain concentration of Nile red dye. We measure this concentration using UV-vis spectroscopy (GENESYS 50 UV–Vis spectrophotometer by ThermoFisher Scientific), from which we can infer the volume of the residual oil since the the initial concentration is known. Note that the UV-Vis spectrophotometer system has a photometric accuracy of ± 0.002 A at 0.5 A and a photometric repeatability of ± 0.001 A at 1 A. Thus, samples with absorption $\le$ 0.004 A fall outside the instrument’s reliable detection limit.

Nile red dye was chosen to stain the displaced oil, as it is suitable for immiscible displacement experiments due to its hydrophobic nature. The ideal initial concentration of Nile red, $C_{init,NR}$, is made to be around 152 $\upmu$ g/mL based on our previous experience^7^. This specific concentration was determined by assuming 60 $\upmu$m to be a representative residual oil film thickness at the end of our experiments, and achieving a Nile red concentration of the resulting THF solution to be approximately 0.8 $\upmu$ g/mL. This level was deemed ideal based on our UV–Vis calibration curve. The oil is mixed with the Nile red dye using an ultrasonic homogenizer for 15 minutes, resulting in a well-mixed, dark orange colored oil with a uniform transparent texture. The mixture is then kept on a magnetic hot plate stirrer operating at 600 revolutions per minute at a temperature of 25 $^{\circ }$C. The Nile red dye used in this study was purchased from TCI Europe, and the organic solvent THF ($> 99\%$ stabilized) is supplied by VWR Chemicals.

To have effective experimental operation, a suitable concentration of Nile red needs to be selected so that the final solution, recovered at the end of the experiment, will have measurable absorbance and not be oversaturated. With this in mind, and the calibration curve, we aimed at obtaining a concentration of Nile red of approximately 0.8 $\upmu$ g/mL in the final residual wall layer. Assuming that an equivalent uniform oil film thickness of 60 $\upmu$m would be representative for several of our experiments, we added 25 mg Nile red to 155 mL mineral oil to arrive at this approximate level of dye in the residual oil at the end of the experiment.

In order to effectively utilize Nile red, a calibration curve needs to be made prior to conducting experiments. Standard procedures and parameters for constructing the calibration curve can be found in our previous publication^7^. The curve, $y = 0.1029x + 0.0008$, reports the absorption intensity as a function of Nile red concentration, which enables us to estimate the residual oil film volume from each experimental run. In summary, with the measured average peak absorbance of 528–530 nm, the concentration of Nile red in THF solution was calculated from the calibration curve. Once the Nile red concentration, $C_{THF,NR}$, is obtained, the volume of residual oil can be calculated based on the known volume of THF solvent ($V_{THF} = 35$ mL) used to submerge the test section and the initial concentration of Nile red in oil using the equation $V_{Res-oil} = C_{THF,NR} \cdot V_{THF}/C_{init,NR}$. A more detailed description of the method used to calculate the residual oil volume from the retrievable test section, given the known initial concentration of Nile red and the volume of THF solvent, can be found in the previous publication^7^.

## Results

### Laminar displacements

**Figure 2:**
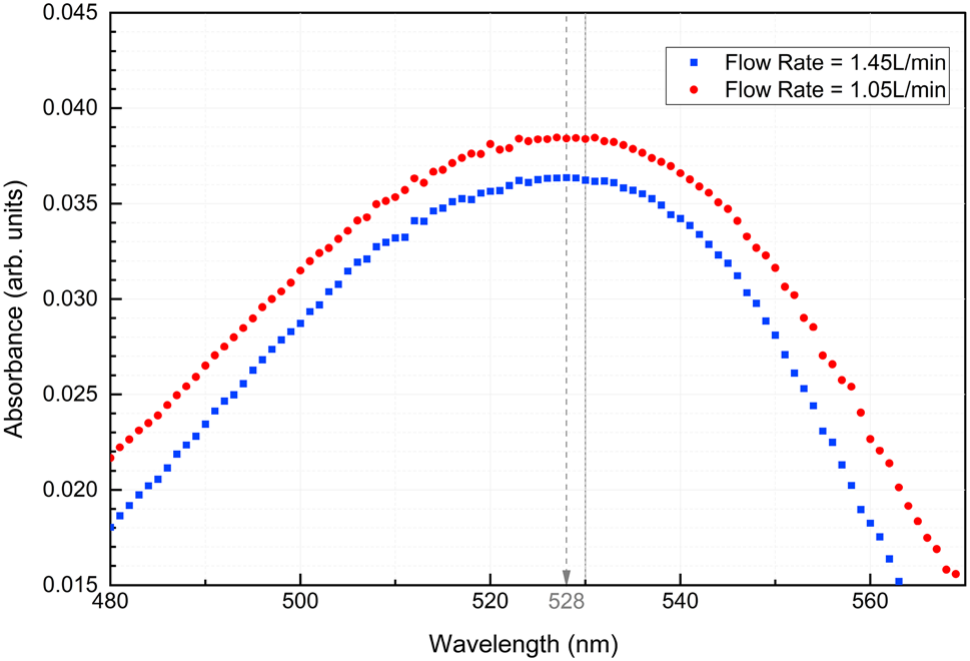
UV–Vis spectra for case 1 and 2.

In our previous work^7^, we reported results from an experimental examination on the efficiency of residual oil film displacement within a horizontal pipe. To extend the scope of that study, we performed new displacement experiments at the same flow rates as used in that study, but now in a vertical pipe configuration, as discussed in connection with Fig. 1. For this purpose, two cases (Case 1 and Case 2 in Table 2) were conducted with flow rates of 1.05 and 1.45 L/min, respectively. Both cases were run with an injected volume of 2 L to ensure that the residual oil film volume was in the asymptotic regime. The UV-Vis absorption spectra for these two laminar cases are shown in Fig. 2. The residual oil volumes calculated from the absorption spectra are presented in Table 4.

**Figure 3:**
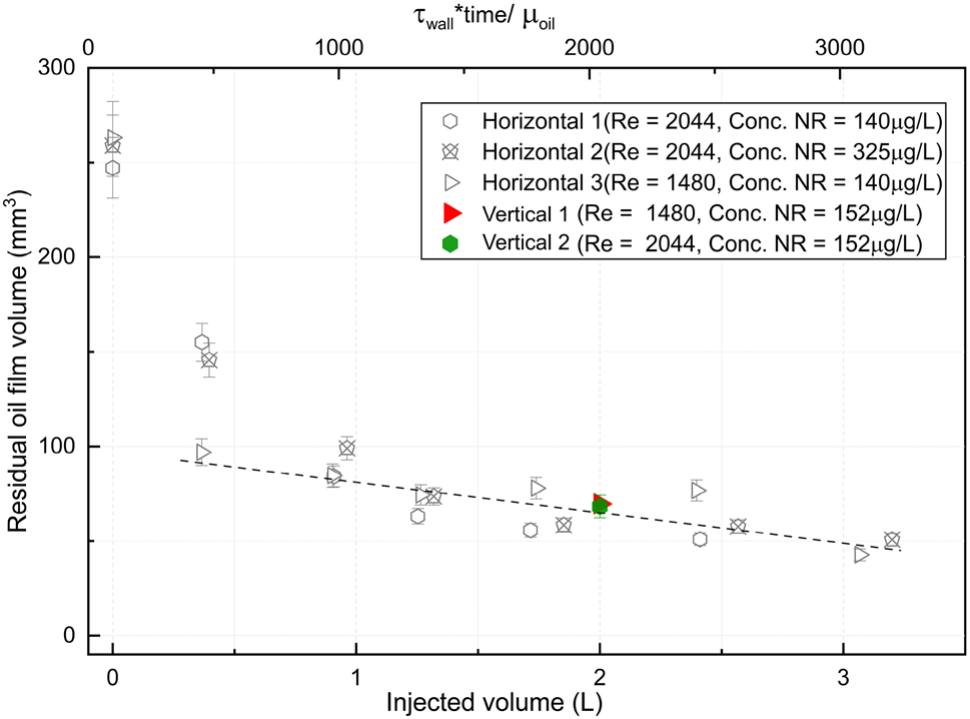
Residual oil film volume for different injected volume.

The calculated residual oil film volumes from our newly measured vertical cases are also plotted, along with all the horizontal cases, as function of injected volume in Fig. 3. The hollow icons represent data from our previous horizontal dataset, and the solid ones represent the new measurements from the vertical setup. A second abscissa is also included in Fig. 3, corresponding to the dimensionless time introduced above, i.e. $T = \tau _w \cdot t / \mu _o$, where we approximate the interfacial shear stress by the wall shear stress, which is $\tau _w = 8\mu _w U / D$ for water. Thus, since $U \cdot t$ is proportional to the injected volume, plotting of the laminar cases in Fig. 3 can done as function of injected volume, or equivalently as function of the dimensionless time. We note that this only holds true for laminar flow, i.e. when $\tau _w \propto U$, and under the assumption that the shear stress at the interface is well approximated by the wall shear stress.

**Table 4:** Calculated residual oil volumes according to Fig. 2.

Case #	Flow rate (L/min)	Nile r concentration ($\upmu$ g/mL)	Residual oil volume (mm$^3$)
1	1.05	0.0052	71.63
2	1.45	0.0054	68.28

As shown in Fig. 3 and Table 4, measurements from vertical cases closely match the laminar flow cleaning curve generated by horizontal cases, indicating the orientation of the pipe (horizontal or vertical) does not have a significant impact on long-term oil film cleaning for laminar flow. This observation is consistent with the thin film/lubrication scaling and evolution equation for the interface thickness, where gravity enters as a higher order term compared to the forcing imposed by the displacing fluid. The percentage difference between the new vertical cases and the best-fit line calculated from the previous work is within the range of percentage error of all cases from the previous work.

Furthermore, our experimental results, as shown in Fig. 3, demonstrate that the wall film thickness (or residual oil volume) measured from experiments appears to collapse onto a single curve when plotted against injected volume, or equivalently the dimensionless time $T = {\tau } \cdot {t} / {\mu }_o$ in the laminar regime. Consequently, we find these results from the laminar displacement experiments to be in line with the theoretical scaling discussed above. Although only narrow range of Reynolds numbers (wall shear stresses) are compared in Fig. 3, we consider the results to support the thin-film model predictions.

### Comparison of displacements under laminar and turbulent conditions

We next compare residual oil volumes for different imposed flow rates (i.e. different Reynolds numbers) in the vertical pipe, covering the cases in Table 2. Results will be presented for injection of either 1 L or 2 L volume of displacing fluid, corresponding to 6.5 and 12.9 times the initial oil volume occupying the pipe before displacement. To ensure consistency and accuracy, each case in Table 2 was conducted five times. This allowed for the identification and exclusion of any outliers, and error bars were calculated accordingly.

**Figure 4:**
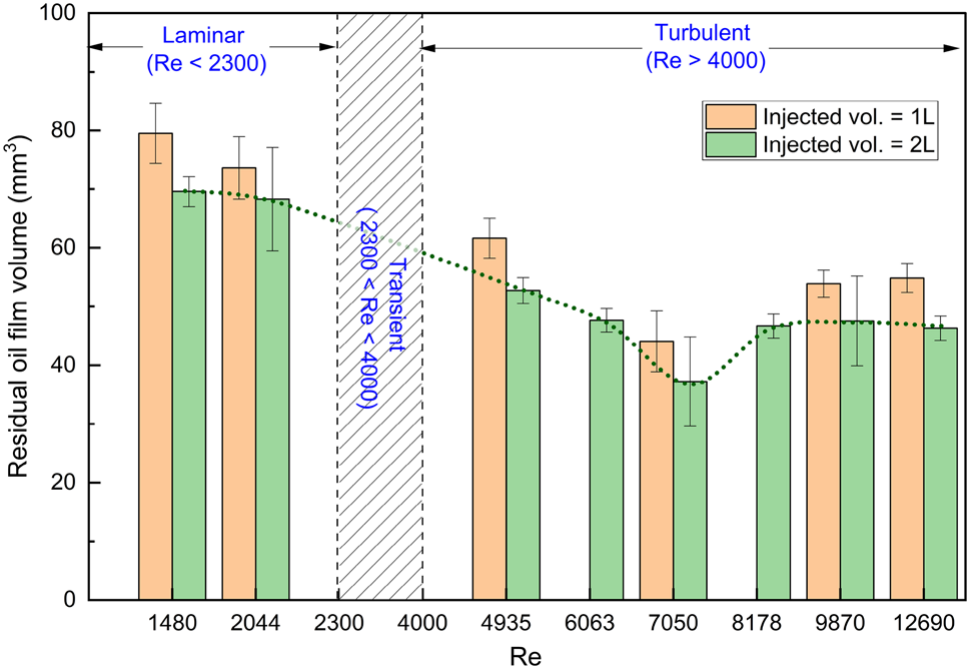
Residual oil film volume for different Reynolds number.

The calculated residual oil film volume for each displacement case is shown in Fig. 4. The results clearly suggest that for equal injected fluid volumes, turbulent flow results in less residual oil volumes. We attribute this observation to the larger mean interfacial stresses in under turbulent conditions, and possibly also relatively significant stress fluctuations that can perturb the interface locally, and trigger instabilities that further improve the wall cleaning.

Furthermore, the results of the displacement experiments shown in Fig. 4 indicate that the Reynolds number (or the associated interfacial shear stress) plays a significant role in the experiments. However, as seen in the figure, the relationship between Reynolds number and residual oil film volume or oil displacement efficiency is not a simple monotonic function, but instead, there exists an optimal range of Reynolds numbers where the cleaning is most efficient, namely at intermediate Reynolds numbers of approximately 7000. The relationship between wall shear stress and Reynolds number is a well-established principle in fluid mechanics. With a fixed injected volume, as the Reynolds number increases, the wall shear stress also increases, promoting more efficient displacement of the oil. However, since these experiments were performed by injecting a fixed volume of displacing fluid, increasing the Reynolds number (by increasing the imposed flow rate) implies a shorter displacement time in these experiments. We go on to explore the combined effect of shear stress and displacement time in the following section.

### Displacement time, shear stress, and their combined effect

To further investigate the combined effect of wall shear stress and displacement time on the displacement process, three cases with low turbulent flow, intermediate turbulent flow, and high turbulent flow were selected to run at different displacement times. Details of the experimental conditions are included in Table 3.

**Figure 5:**
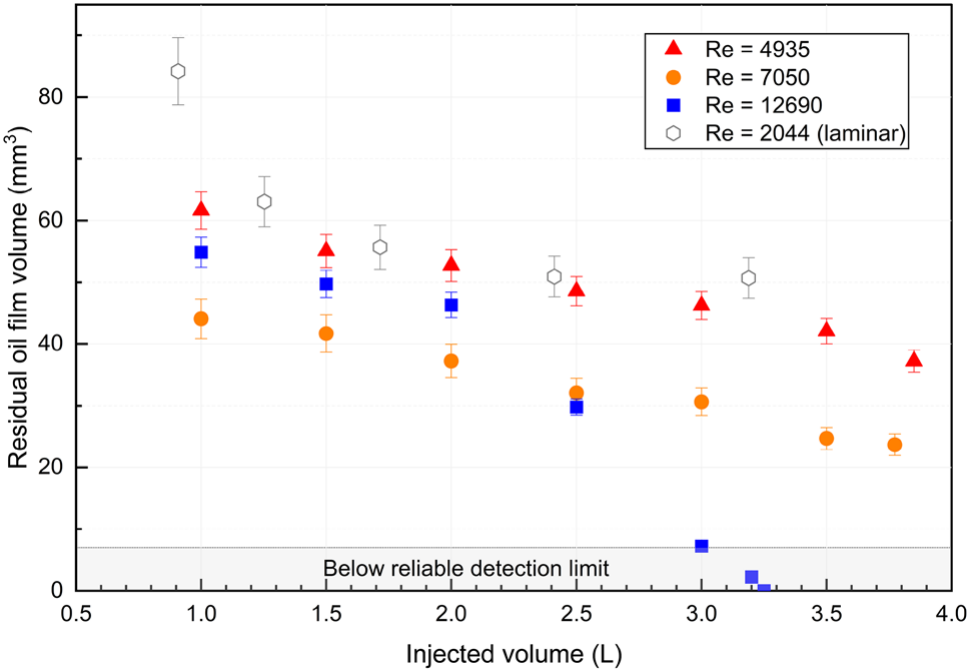
Residual oil film volume vs. injected fluid volumes for different Reynolds number.

**Figure 6:**
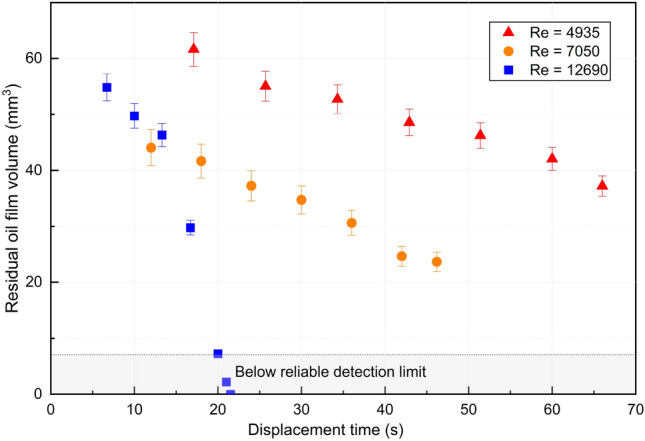
Residual oil film volume vs. displacement time for different Reynolds number.

**Figure 7:**
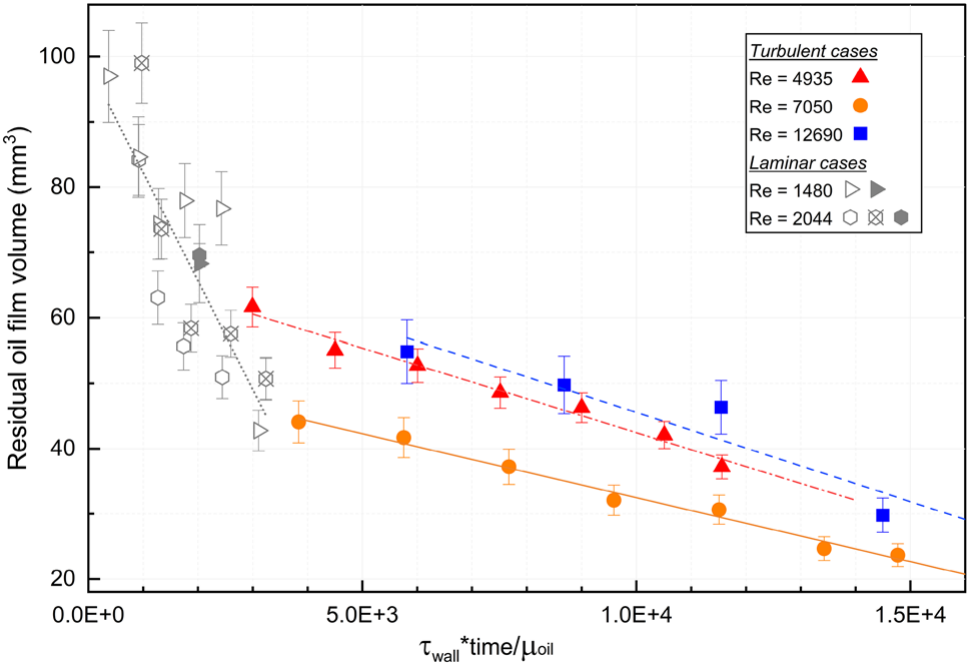
Residual oil film volume as a function of dimensionless time. The open symbols (laminar displacement conditions) correspond to experiments performed in a horizontal pipe, presented in Ref.^7^. Filled symbols correspond to new experiments in a vertical pipe configuration.

In Figs. 5, 6, and 7, the residual oil film volumes were separately plotted against displacement time and injected fluid volumes, as well as against the combined effect of wall shear stress and displacement time, represented by the dimensionless time $T = \tau _w\cdot t /\mu _o$. We set $\tau _w = 8 \mu _w U / D$ for experiments corresponding to laminar Reynolds numbers ($Re < 2300$), and take $\tau _w = \rho U^2 f_D / 8$, with $f_D$ given by Eq. (2) for turbulent cases ($Re > 4000$). The dimensionless time captures the balance between the product of applied shear stress and displacement time (equivalent to a momentum divided by interfacial surface area) to that of the oil viscosity.

When considering the residual oil volume as a function of displacement time for the different turbulent regimes in Figs. 5 and 6, we observe that the low and intermediate turbulent regimes exhibit similar trends. Specifically, for small injected volumes (less than 2.5 L), the intermediate turbulent regime showed a better cleaning efficiency than both the high and low turbulent regimes. This result indicates that the intermediate regime is most efficient for achieving cleaning using small injected volumes.

For longer displacement times or larger volumes, the highly turbulent regime results in a much improved cleaning rate, as shown in Figs. 5 and 6. As the displacement time or injected volume increases, the combined effects of large interfacial shear stresses and turbulent fluctuations result in effective cleaning. Importantly, the high turbulent regime is also the only case where the residual oil film volume can be reduced to a level that is undetectable by UV–Vis, indicating that it is the most effective regime for thorough cleaning.

Finally, the measurements of residual oil volume as function of dimensionless time, Fig. 7, suggest that the low and highly turbulent regimes collapse onto a single curve, while the intermediate regime results in a smaller residual volume at the same dimensionless time. The three turbulent regimes all appear to produce a linearly decreasing trend in residual oil volume as function of dimensionless time. The linear fitting details are shown in Table 5 for completeness.

**Table 5:** Linear regression results.

*Re*	Linear fit ($T = \tau _w \cdot t / \mu _{o}$)	Adj. R-square
4935	$V_{oil} = -0.00259\cdot T + 68.341$	0.977
7050	$V_{oil} = -0.00196 \cdot T + 52.090$	0.983
12,690	$V_{oil} = -0.00273 \cdot T + 72.798$	0.821
Laminar	$V_{oil} = -0.0166 \cdot T + 98.795$	0.656

## Discussion

### Turbulent wall shear stress

Regarding the removal of the oil film, our work demonstrates that turbulent flow produces more promising results. We note that measured residual oil volumes for the turbulent displacement experiments ranged up to approximately 60 mm$^3$. Assuming a uniform oil film along the 7 cm long test section, this volume is equivalent to a uniform film thickness up to approximately 20 $\upmu$m. Fully-developed turbulent pipe flow will exhibit a viscous sublayer adjacent to the wall, and the thickness of this layer is often assumed to be $\delta _{visc} = 5 \mu _w / \sqrt{\rho _w \tau _w}$ for smooth-walled pipes^15^. Thus, the thickness of this sublayer decreases with increasing wall shear stress, i.e. increasing Reynolds number, and we estimate viscous sublayer thickness of approximately 221 $\upmu$m at $Re = 4935$ to 98 $\upmu$m at $Re = 12{,}690$. This suggests that the residual oil film thickness after injection of 1 L displacing fluid, or more, is of the same order, or less, than the viscous sublayer in fully-developed turbulent pipe flow. Since the residual film thickness is so thin, we consider it reasonable to assume that the shear stress is constant in the vicinity of the interface, and that Eq. (2) is a relevant thin-film approximation for the wall film removal. This also confirms the scaling adopted in Fig. 7. We also note that for laminar displacement cases, the test section is sufficiently far downstream from the inlet, so that the residual oil volume is within the laminar boundary layer adjactent to the wall^16^.

Based on our observations, both displacement time and Reynolds number play important roles in the efficiency of clean-in-place procedures with turbulent flow. However, as mentioned in the literature^17,18^, the effect of displacement time is limited and not as dominant as the effects brought about by the physical force introduced by turbulent flow. In turbulent flow, there is a rapid increase in wall shear stress and its fluctuation rate. Turbulent flows are characterized by fluctuations in velocity and pressure in both space and time, and the magnitude of these fluctuations typically increase with the mean wall shear stress; Alfredsson et al. reported typical values of streamwise stress fluctuations of about 40% of the mean wall shear stress, and spanwise fluctuations of approximately 20%^19^. It was also observed that these fluctuations are practically constant within the viscous sublayer,^19^.

### Level of turbulent for oil film removal applications

Regarding the removal of oil film, although our work demonstrates that turbulent flow produces more promising results, it may not always be the optimal choice in practical applications. The operation can become more complicated when working with complex fluids, such as yield stress fluids^20^, making it challenging to achieve fully turbulent flow. For industrial applications, which often involve more complex geometries than a simple pipe, such as in the case of eccentric annuli, the eccentricity can adversely affect the ability to create turbulent flow throughout the entire annulus, resulting in turbulent flow on only one side of the annulus^21^.

Based on our observations, intermediate turbulent flow appears to have better cleaning efficiency when displacement time is limited. However, for longer displacement times, high turbulent flow seems to be the most effective for thorough cleaning. In real-world applications where there are significant density and viscosity differences between fluids or when scaling up to larger pipes is required, selecting the appropriate level of turbulence is critical. The intermediate turbulent flow might be a more suitable option than a high turbulent regime as it is easier to achieve and can provide better stability while reducing potential problems associated with high turbulent flow.

## Conclusion

In this study, a comprehensive experimental investigation was conducted to examine the influence of flow regimes, including laminar and various levels of turbulence, on the efficiency of residual oil film displacement within a pipe. The findings provide significant insights into the oil film displacement process, with implications for designing advanced pipe-cleaning strategies across a range of industrial applications.

One of the important discoveries is the relationship between the onset of turbulence and the efficiency of residual oil film removal. As the flow transitioned from laminar to turbulent, a substantial decrease in the volume of residual oil film was observed. Our hypothesis is that this increased removal efficiency with turbulent flow is attributed to the fluctuation components introduced by turbulence, which significantly enhanced the wall shear stress. Published results suggest that the magnitudes of streamwise and spanwise stress fluctuations are approximately 40% and 20% of the mean wall shear, respectively. This significant increase in wall shear stress due to turbulent fluctuations is a crucial factor in improved cleaning efficiency.

Interestingly, we observed the existence of an optimal range of Reynolds numbers for efficient oil removal with turbulent flow, particularly when the injected volume of fluid was relatively limited. For injected volume below 2.5 L (< 15 of the oil volume) , the optimal range of *Re* occurred between 7000 and 8000. Lower fixed injected volumes (1 L,  6 of the oil volume) also produced similar trends, lending confidence to this finding. However, the reason why this range is superior to higher Reynolds numbers remains unclear. Future studies employing turbulent flow modeling methods, such as Large Eddy Simulation (LES), could deepen our understanding of this phenomenon.

Additionally, we explored the relationship between wall shear stress, displacement time, and their coupling effect on residual oil film removal. Experiments with varying injected volumes and times were conducted for low, intermediate, and high turbulent flows. The intermediate turbulent regime demonstrated greater cleaning efficiency for small injected fluid volumes. In contrast, the high turbulent regime was most effective for thorough cleaning with longer displacement times or larger injected volumes. This observation is in agreement with industry guidelines that recommend a certain, minimum contact time to ensure full cleaning of the wall, i.e. shear-driven instabilities of the interface do not occur instantaneously, but require a certain minimum time to develop and act. When considering the combined impact of wall shear stress and time, the intermediate turbulent regime yielded the most favorable cleaning efficiency.

This study offers valuable insights into the factors governing residual oil removal efficiency during oil film displacement processes. The experimental dataset on residual oil film following laminar or turbulent fluid displacements expands the existing knowledge base and can serve as benchmark data for developing more effective experimental methods and numerical models. Future work could involve employing turbulent modeling techniques, such as large eddy simulation (LES), to generate data and compare it with the experimental results and correlation predictions, as only a rough estimation of turbulent fluctuation values has been conducted in this study (Supplementary Informations).

### Supplementary Information

Supplementary Information 1.

Supplementary Information 2.

Supplementary Information 3.

Supplementary Information 4.

## Data Availability

Additional datasets generated during and/or analysed during the current study are available from the corresponding author on reasonable request.
