# Supplementary material for: Experimental investigation of laminar and turbulent displacement of residual oil film
Source: Sci Rep. 2023 Nov 30;13:21120. doi: 10.1038/s41598-023-48563-x (PMC10689780; doi:10.1038/s41598-023-48563-x)
Supplement: Supplementary file 1 — Supplementary Information 1. [file 41598_2023_48563_MOESM1_ESM.pdf]

Fig2

X = wavelength (nm)
Y1 = Absorbance (arb.unit), 1.45L/min
Y2 = Absorbance (arb.unit), 1.05L/min

X	Y1	Y2
600	-0.0022	0.0048
599	-0.0019	0.0053
598	-0.0019	0.0051
597	-0.0015	0.0047
596	-0.0017	0.0050
595	-0.0015	0.0049
594	-0.0013	0.0056
593	-0.0014	0.0057
592	-0.0016	0.0055
591	-0.0007	0.0061
590	-0.0009	0.0061
589	-0.0004	0.0059
588	-0.0008	0.0060
587	0.0000	0.0064
586	0.0003	0.0070
585	0.0003	0.0074
584	0.0012	0.0073
583	0.0012	0.0072
582	0.0013	0.0080
581	0.0021	0.0087
580	0.0021	0.0090
579	0.0027	0.0097
578	0.0032	0.0101
577	0.0039	0.0105
576	0.0043	0.0112
575	0.0051	0.0116
574	0.0055	0.0124
573	0.0062	0.0130
572	0.0072	0.0135
571	0.0077	0.0138
570	0.0085	0.0144
569	0.0095	0.0156
568	0.0105	0.0158
567	0.0111	0.0169
566	0.0122	0.0175
565	0.0131	0.0184
564	0.0141	0.0192
563	0.0152	0.0201
562	0.0164	0.0214
561	0.0175	0.0221
560	0.0183	0.0227
559	0.0190	0.0241
558	0.0203	0.0254
557	0.0213	0.0258
556	0.0225	0.0266
555	0.0231	0.0270
554	0.0244	0.0285

553	0.0253	0.0290
552	0.0261	0.0302
551	0.0271	0.0307
550	0.0281	0.0317
549	0.0289	0.0323
548	0.0298	0.0327
547	0.0303	0.0333
546	0.0312	0.0341
545	0.0319	0.0347
544	0.0323	0.0351
543	0.0329	0.0356
542	0.0334	0.0359
541	0.0339	0.0363
540	0.0342	0.0366
539	0.0344	0.0370
538	0.0349	0.0372
537	0.0353	0.0374
536	0.0355	0.0377
535	0.0357	0.0379
534	0.0358	0.0381
533	0.0361	0.0382
532	0.0362	0.0383
531	0.0362	0.0385
530	0.0362	0.0384
529	0.0364	0.0384
528	0.0364	0.0384
527	0.0364	0.0385
526	0.0363	0.0384
525	0.0363	0.0384
524	0.0361	0.0383
523	0.0362	0.0384
522	0.0360	0.0379
521	0.0357	0.0378
520	0.0357	0.0381
519	0.0356	0.0376
518	0.0352	0.0376
517	0.0353	0.0374
516	0.0351	0.0371
515	0.0348	0.0368
514	0.0346	0.0367
513	0.0341	0.0361
512	0.0341	0.0363
511	0.0332	0.0357
510	0.0332	0.0354
509	0.0330	0.0351
508	0.0327	0.0350
507	0.0321	0.0343
506	0.0319	0.0341
505	0.0315	0.0336
504	0.0308	0.0331
503	0.0304	0.0327
502	0.0297	0.0324
501	0.0293	0.0320
500	0.0287	0.0315

499	0.0283	0.0309
498	0.0279	0.0304
497	0.0274	0.0300
496	0.0268	0.0296
495	0.0263	0.0290
494	0.0256	0.0285
493	0.0250	0.0280
492	0.0247	0.0275
491	0.0241	0.0270
490	0.0235	0.0265
489	0.0228	0.0259
488	0.0224	0.0254
487	0.0219	0.0250
486	0.0212	0.0245
485	0.0206	0.0239
484	0.0202	0.0235
483	0.0197	0.0231
482	0.0192	0.0227
481	0.0187	0.0222
480	0.0180	0.0217
479	0.0175	0.0211
478	0.0170	0.0208
477	0.0165	0.0204
476	0.0160	0.0199
475	0.0156	0.0195
474	0.0151	0.0190
473	0.0147	0.0186
472	0.0142	0.0183
471	0.0137	0.0178
470	0.0133	0.0174
469	0.0127	0.0171
468	0.0123	0.0166
467	0.0118	0.0162
466	0.0115	0.0159
465	0.0111	0.0155
464	0.0107	0.0152
463	0.0103	0.0148
462	0.0098	0.0144
461	0.0095	0.0141
460	0.0092	0.0138
459	0.0090	0.0135
458	0.0086	0.0132
457	0.0082	0.0129
456	0.0079	0.0127
455	0.0075	0.0122
454	0.0072	0.0120
453	0.0070	0.0118
452	0.0067	0.0116
451	0.0064	0.0114
450	0.0061	0.0112
