# Supplementary material for: Experimental investigation of laminar and turbulent displacement of residual oil film
Source: Sci Rep. 2023 Nov 30;13:21120. doi: 10.1038/s41598-023-48563-x (PMC10689780; doi:10.1038/s41598-023-48563-x)
Supplement: Supplementary file 2 — Supplementary Information 2. [file 41598_2023_48563_MOESM2_ESM.pdf]

#Fig3

X1 = Inject Vol.(L)
X2 = Dimensionless time
Y = Oil film vol. (mm^3)

Horizontal 1

X1	X2	Y	Error bar (12.94%*Y)
0.0	0	247.17	15.99
0.4	371	155.05	10.03
0.9	920	84.19	5.45
1.3	1269	63.07	4.08
1.7	1738	55.66	3.60
2.4	2443	50.93	3.29

Horizontal 2

X1	X2	Y	Error bar (12.43%*Y)
0.0	0	258.79	16.08
0.4	401	145.61	9.05
1.0	974	99.02	6.15
1.3	1335	73.59	4.57
1.9	1876	58.40	3.63
2.6	2600	57.56	3.58
3.2	3241	50.69	3.15

Horizontal 3

X1	X2	Y	Error bar (14.55%*Y)
0.0	0	263.06	19.14
0.4	370	96.99	7.06
0.9	914	84.63	6.16
1.3	1281	74.38	5.41
1.7	1761	77.94	5.67
2.4	2428	76.74	5.58
3.1	3106	42.77	3.11

Vertical 1

X1	X2	Y	Error bar (14.55%*Y)
2.0	2026	69.58	1.79

Vertical 2

X1	X2	Y	Error bar (8.79%*Y)
2.0	2026	68.28	6.00
