# Supplementary material for: Experimental investigation of laminar and turbulent displacement of residual oil film
Source: Sci Rep. 2023 Nov 30;13:21120. doi: 10.1038/s41598-023-48563-x (PMC10689780; doi:10.1038/s41598-023-48563-x)
Supplement: Supplementary file 3 — Supplementary Information 3. [file 41598_2023_48563_MOESM3_ESM.pdf]

#Fig4

X = Re
Y1 = Oil film volume (mm^3), 1L inject Vol.
Y1 = Oil film volume (mm^3), 2L inject Vol.

X	Y1	Y1 Error bar	Y2	Y2 Error bar
1480	79.50	5.14	69.58	2.58
2044	73.63	5.34	68.28	8.79
4935	61.64	3.41	52.72	2.21
6063	NAN	NAN	47.65	2.00
7050	44.07	5.19	37.22	7.60
8178	NAN	NAN	46.68	2.06
9870	53.89	2.30	47.54	7.66
12690	54.85	2.43	46.32	2.06
