# Supplementary material for: Experimental investigation of laminar and turbulent displacement of residual oil film
Source: Sci Rep. 2023 Nov 30;13:21120. doi: 10.1038/s41598-023-48563-x (PMC10689780; doi:10.1038/s41598-023-48563-x)
Supplement: Supplementary file 4 — Supplementary Information 4. [file 41598_2023_48563_MOESM4_ESM.pdf]

#Fig5-7

X1 = Inject Vol.(L)
X2 = Contact time (t)
X3 = Dimensionless time
Y = Oil film vol. (mm^3)

Re = 4935

X1	X2	X3	Y	Error bar (9.8%*Y)
1.0	17	2995	61.64	3.02
1.5	26	4501	55.04	2.70
2.0	34	6007	52.72	2.58
2.5	43	7513	48.57	2.38
3.0	51	9002	46.24	2.27
3.5	60	10508	42.07	2.06
3.85	66	11559	37.19	1.82

Re = 7050

X1	X2	X3	Y	Error bar (7.28%*Y)
1.0	12	3836	44.07	3.21
1.5	18	5754	41.68	3.03
2.0	24	7672	37.22	2.71
2.5	30	9590	32.07	2.33
3.0	36	11508	30.62	2.23
3.5	42	13426	24.66	1.79
3.77	46	14769	23.66	1.72

Re = 12690

X1	X2	X3	Y	Error bar (8.87%*Y)
1.0	7	5816	54.85	2.43
1.5	10	8680	49.72	2.21
2.0	13	11545	46.32	2.06
2.5	17	14496	29.78	1.32
3.0	20	17360	7.23	0.32
3.2	21	18228	2.22	0.10
3.25	22	18662	0.00	0.00

Re = 2044 (laminar)

X1	Y	Error bar (6.45%*Y)
0.4	155.05	10.00
0.9	84.19	5.43
1.3	63.07	4.07
1.7	55.66	3.59
2.4	50.93	3.28
3.2	50.69	3.27
